# HPV-associated head and neck cancer is characterized by distinct profiles of CD8^+^ T cells and myeloid-derived suppressor cells

**DOI:** 10.1007/s00262-023-03571-8

**Published:** 2023-11-29

**Authors:** Benjamin A. Kansy, Tim P. Wehrs, Kirsten Bruderek, Yu Si, Sonja Ludwig, Freya Droege, Pia Hasskamp, Uta Henkel, Nina Dominas, Thomas K. Hoffmann, Peter A. Horn, Martin Schuler, Thomas C. Gauler, Monika Lindemann, Stephan Lang, Agnes Bankfalvi, Sven Brandau

**Affiliations:** 1 Research Division, Department of Otorhinolaryngology, West German Cancer Center, University Duisburg-Essen, University Hospital Essen, Hufelandstrasse 55, 45122 Essen, Germany; 2 Department of Otorhinolaryngology, Head and Neck Surgery, University Hospital Mannheim, Mannheim, Germany; 3 Department of Otorhinolaryngology, University Hospital Ulm, Ulm, Germany; 4 Institute for Transfusion Medicine, University Duisburg-Essen, University Hospital Essen, Essen, Germany; 5 Department of Medical Oncology, University Duisburg-Essen, University Hospital Essen, Essen, Germany; 6 German Cancer Consortium (DKTK), Partner Site University Hospital Essen, Essen, Germany; 7 Department of Radiation Oncology, University Duisburg-Essen, University Hospital Essen, Essen, Germany; 8 Institute of Pathology, University Duisburg-Essen, University Hospital Essen, Essen, Germany; 9 Department of Otolaryngology, Head and Neck Surgery, Sun Yat-Sen Memorial Hospital, Sun Yat-Sen University, Guangzhou, China

**Keywords:** HPV, MDSC, CD8^+^ T cells, Head and neck cancer, Oropharyngeal cancer

## Abstract

Patients with HPV^−^-localized head and neck cancer (HNC) show inferior outcomes after surgery and radiochemotherapy compared to HPV-associated cancers. The underlying mechanisms remain elusive, but differences in immune status and immune activity may be implicated. In this study, we analyzed immune profiles of CD8^+^ T cells and myeloid-derived suppressor cells (MDSC) in HPV^+^ versus HPV^−^ disease.

The overall frequency of CD8^+^ T cells was reduced in HNC versus healthy donors but substantially increased after curative therapy (surgery and/or radiochemotherapy). In HPV^+^ patients, this increase was associated with significant induction of peripheral blood CD8^+^/CD45RA^−^/CD62L^−^ effector memory cells. The frequency of HPV-antigen-specific CD8^+^ cells was low even in patients with virally associated tumors and dropped to background levels after curative therapy. Pre-therapeutic counts of circulating monocytic MDSC, but not PMN-MDSC, were increased in patients with HPV^−^ disease. This increase was accompanied by reduced fractions of terminally differentiated CD8^+^ effector cells. HPV^−^ tumors showed reduced infiltrates of CD8^+^ and CD45RO^+^ immune cells compared with HPV^+^ tumors. Importantly, frequencies of tumor tissue-infiltrating PMN-MDSC were increased, while percentages of Granzyme B^+^ and Ki-67^+^ CD8 T cells were reduced in patients with HPV^−^ disease.

We report differences in frequencies and relative ratios of MDSC and effector T cells in HPV^−^ HNC compared with more immunogenic HPV-associated disease. Our data provide new insight into the immunological profiles of these two tumor entities and may be utilized for more tailored immunotherapeutic approaches in the future.

**Supplementary Information:**

The online version contains supplementary material available at 10.1007/s00262-023-03571-8.

## Introduction

Over the past decades, head and neck cancer (HNC), the worldwide 6th most common cancer, has experienced a significant change due to the identification of human papillomavirus (HPV)-associated disease, a molecular entity that is clearly distinct from HPV^−^ HNC [1]. In the oropharynx, the high-risk subtype HPV16 is–by far–the most frequent HPV subtype associated with the disease. Despite the fact that HPV^+^ HNC is usually diagnosed at an advanced nodal infestation, therapeutic response and survival is significantly better in comparison with HPV^−^ HNC patients [2–4]. In some studies, the favorable prognosis was attributed to an increased sensitivity of HPV^+^ HNC to radiation [5]. However, recent evidence suggests that differences in immune profiles and response may contribute to the improved prognosis of HPV^+^ HNC patients [4, 6, 7]. In the peripheral blood, HNC is characterized by profound signs of immunosuppression associated with reduced T cells counts and induction of regulatory T cells as well as myeloid-derived suppressor cells (MDSC) [8–11]. Although the prognostic relevance of circulating CD8^+^ T cells is less clear and demands further investigation, some studies already reported on the identification of CD8^+^ T cell subsets, which were associated with a more favorable outcome [12]. Therefore, further investigations exploring potential differences in T cell subsets of HPV^+^ and HPV^−^ HNC patients are required, including functionality, antigen specificity, phenotypes, and—of utmost importance—possible causes for these differences.

Over the past decade, MDSC, which are induced in HNC and many other types of cancer [8], have emerged as important regulators of CD8^+^ T cell-mediated immunity. MDSC are a heterogeneous group of myeloid cells, and several mechanisms have been identified how these cells regulate and inhibit the activity of other immune cells, particularly T cells [13, 14]. Originally identified in mice [15], more recently, MDSC and subsets thereof have also been described in humans [16–18].

It was the aim of this study to investigate the frequency and subset distribution of CD8^+^ T cells and MDSC in patients with primary HNC. We focused our detailed longitudinal and comparative analysis of the CD8^+^ T cell compartment on a comparative analysis of HPV^+^ versus HPV^−^ HNC and hypothesized that differences in MDSC and CD8^+^ T cell subsets exist between these HNC subtypes. Our findings suggest different baseline levels and dynamics of MDSC and T cell phenotypes in HPV^−^ versus HPV^+^ HNC, which may contribute to differences in clinical outcome.

## Materials and methods

### Patients and control subjects and HLA/HPV testing

Blood samples were taken from 89 head and neck cancer patients before the onset of therapy (pre-therapy sample) and again beginning from the first tumor-free check-up (post-therapy samples). Pre-therapy blood samples were used for HLA typing (performed at the Institute for Transfusion Medicine, University Hospital Essen). HPV status was determined in primary tumor material by an HPV family gene probe (INFORM HPV III Family 16, Roche Diagnostics, Mannheim, Germany) followed by a (PGMY09/11 & GP5^+^/6^+^) nested PCR (performed by the Institute of Pathology). In addition, surplus tumor tissue samples were stained for pRb and p16 (see below). This staining pattern was used as a surrogate marker for HPV RNA-positivity according to a method described by Holzinger et al. [19]. Blood samples from 18 healthy control donors were taken and analyzed accordingly. Experiments were approved by the local ethics committee, and informed written consent was obtained from each individual.

### Mononuclear cell isolation and cryopreservation

Peripheral venous blood was collected in sodium-citrate S- Monovettes (9NC) (Sarstedt, Nümbrecht, Germany). For isolation of mononuclear cells (MNCs) blood was diluted (1:1, *v/v*) with phosphate-buffered saline (PBS; ThermoFisher scientific, Karlsruhe, Germany) and subjected to density gradient centrifugation using Biocoll Separation Solution (Merck, Darmstadt, Germany). The MNC fraction was collected into a new tube and washed several times with PBS. For longitudinal T cell analysis, MNC were stored in 90% fetal calf serum (FCS; Merck) with 10% CryoSure-dimethyl sulfoxide (DMSO; Wak Chemie Medical, Bettingen, Germany) in liquid nitrogen. Cells were thawed in FCS (10:1, *v/v*), centrifuged and resuspended in RPMI 1640 (Invitrogen) supplemented with 5% human serum (Institute for Transfusion Medicine) and 1% penicillin/streptomycin (ThermoFisher scientific). After thawing, the cells were allowed to rest at 4 × 10^6^ cells/mL in medium at 37° C/5% CO_2_ overnight. Cell numbers were determined with a Casy Cell Counter (OMNI Life Science, Bremen, Germany). For MDSC analysis fresh blood samples were analyzed [20]. Of the 89 HNSCC patients, 74 were stained for HLA-A2 status. The HLA-A2 frequency was 42% in HPV^+^ patients and did not differ from frequencies in HPV^−^ patients (42%).

### Flow cytometric analysis of T cell phenotype

0.8–1 × 10^6^ MNCs were stained with antibodies for 30 min at 4° C in PBS with 0.1% bovine serum albumin (BSA, Applichem, Darmstadt, Germany), followed by live/dead staining with fixable viability dye eFluor-780 (eBioscience, Frankfurt, Germany) in PBS for additional 30 min at 4° C. Cells were resuspended in 200 µL PBS + 0.1% BSA for flow cytometry. The following antibodies were used: CD3-FITC (clone BW264/56, Miltenyi Biotec, Bergisch–Gladbach, Germany); CD8-V500 (clone RPA-T8), CD45RO-FITC (clone UCHL1), CD62L-V450 (clone DREG-56), (both BD Bioscience, Heidelberg, Germany); CD45RA-PE-Cy7 (clone HI10 Biolegend, Koblenz, Germany); CCR7-PE (clone 150503, BioTechne, Wiesbaden, Germany); PD-1-APC (clone eBioJ105),TIM-3-PerCP-eFluor710 (clone F38-2E2, both eBioscience). Cells were analyzed on a FACSCanto II using the Diva software 6.0 (BD Bioscience).

### Analytical flow cytometry of MDSC

For characterization of MDSC subsets the following antibodies were used: CD66b FITC (clone 80H3, Beckman Coulter, Krefeld, Germany), CD33 PE (clone WM53), HLA-DR APC (clone G46-6), CD16 PE-Cy7 (clone 3G8), CD11b APC-Cy7 (all BD Bioscience, Heidelberg, Germany), and CD14 PerCP-Cy5.5 (clone HCD14). MDSC in HNSCC cancer patients were defined as formerly described in Lang et al. [21] as CD14^+^/CD66b^−^/CD33^high^/HLA-DR^low^ M-MDSC, CD14^−^/CD66b^+^/CD33^dim^/HLA-DR^low^ or HLA-DR^negative^ PMN-MDSC and CD14^−^/CD66b^−^/CD33^dim^/HLA-DR^low^ or HLA-DR^negative^ e-MDSC.

### Peptide-MHC class I complex analysis (Dextramer)

1–3 × 10^6^ MNCs were stained with CD3-FITC, CD8-V500, fixable viability dye eFluor^®^-780 and either negative control or HPV E7_11–20_ Dextramer-PE. The staining procedure was performed according to the manufacturer’s protocol. The fixable viability dye was used after antibody and Dextramer staining. Negative control Dextramer-PE and HPV E7_11–20_ (YMLDLQPETT) Dextramer-PE were obtained from Immudex (Copenhagen, Denmark).

### Interferon gamma (IFN-γ) enzyme-linked immunospot assay (ELISpot)

1 × 10^5^ MNCs were seeded into 96-well U bottom plates (Greiner bio-one, Frickenhausen, Germany) in RPMI medium supplemented as above with or without 6 nmol/mL each of Peptivator HPV16 E6 & E7 (Miltenyi Biotec, Bergisch–Gladbach, Germany) for 24 h. The assay was then transferred to IFN-*γ*-coated MultiScreen-HTS-IP (Merck Millipore, Darmstadt, Germany) for another 48 h. IFN-*γ* capture (clone 1-D1K) and biotinylated detection (clone 7-B6-1) antibodies were purchased from Mabtech AB (Cologne, Germany); ExtrAvidin®–Alkaline Phosphatase and BCIP/NBT solution was purchased from Sigma-Aldrich (Taufkirchen, Germany). Spots were counted by an AID iSpot FluoroSpot Reader System with the EliSpot 6.0 iSpot software (AID Diagnostika, Straßberg, Germany). Phytohemagglutinin-L (PHA, Sigma-Aldrich, Taufkirchen, Germany) was utilized as a positive control.

### Multiple Immunofluorescence staining of HNC tumor tissue

Five-μm-thick sections from fresh frozen Tissue-Tek^®^ O.C.T.™ Compound (Sakura Finetek, Staufen, Germany) embedded tumor tissue were prepared. Sections were fixed for 15min with Cytofix/Cytoperm Kit (BD Bioscience, Heidelberg, Germany) and incubated with the unconjugated primary antibody rabbit anti-human LOX-1 (Abcam, Cambridge UK) and simultaneously with mouse anti-human Granzyme B (clone GB11, LS Bio, Shirely, USA) or mouse anti-human Ki67 (clone MIB-1, Agilent Technologies, Waldbronn, Germany) in a humid chamber at 4 ℃ overnight. Subsequently, samples were incubated with goat anti-mouse IgG (H + L) Alexa Flour 405 and donkey anti-rabbit IgG (H + L) Alexa 546 (both, ThermoFisher scientific) for 45 min followed by 60-min mouse anti-human CD66b FITC (clone 80H3, Beckmann coulter, Krefeld, Germany) and CD8 Alexa Flour 647 (clone LT8, Bio-Rad, München, Germany). Samples were mounted with eBioscience Fluoromount-G (ThermoFisher scientific) and analyzed by fluorescence microscopy using automated Zeiss Axio Observer and Zen blue software (Carl Zeiss, Jena Germany) at the Imaging Center Essen (Service Core Facility of the Faculty of Medicine of the University Duisburg-Essen, Germany). Samples were analyzed for frequency of CD66b^+^/LOX1^+^ and CD8^+^/Granzyme B^+^ or CD8^+^/Ki67^+^ cells within tumor or stroma with Definiens Tissue Studio Software (Definiens, München, Germany). For analysis of CD66b^+^/LOX1^+^ density the following reagents were used: rabbit anti-human LOX-1, donkey anti-rabbit IgG (H + L) Alexa 546 and mouse anti-human CD66b FITC followed by 15 min 4′,6-diamidino-2-phenylindol (DAPI, BioLegend, Koblenz, Germany) nucleus counterstaining.

### Preparations of tumor microarrays (TMAs)

Formalin-fixed and paraffin-embedded tumor tissue from HNC patients was cut into 1.5-µm-thick whole tissue sections. Hematoxylin and eosin (H&E)-stained sections were used to identify and mark representative tumor regions by an experienced pathologist (AB). Marked tumor regions were punched out (3 mm diameter) and transferred into empty recipient blocks (spaced 1 mm apart). Tissue punches from kidney, liver, and healthy mucosa were added as controls. Blocks were sealed, 3-µm-thick sections were cut, transferred on slides (SuperFrost, R. Langenbrinck Labor- und Medizintechnik, Emmendingen, Germany), and dried over night at 60 °C.

### Immunohistochemistry (IHC) and scoring system

pRB and p16^INK4a^ were stained on whole tissue sections. The other antibodies were stained on TMAs. p16^INK4a^ was stained with a Dako Autostainer (DakoCytomation, Hamburg, Germany). For the other antibodies, sections were deparaffinized and rehydrated. Heat-induced epitope retrieval (HIER) with citrate buffer (Invitrogen) was performed, followed by inactivation of endogenous peroxidase (Dako, Hamburg, Germany). Sections were stained with target antibody, followed by Horseradish peroxidase-coupled (HRPO) secondary and tertiary antibody staining. Sections were counterstained with Shandon hematoxylin (Thermo Scientific, Bonn, Germany). Following antibodies were used for IHC: pRB (clone 1F8, Leica, Wetzlar, Germany), CD45RO (clone UCHL1, abcam, Cambridge, UK); CD8β (clone F-5, Santa Cruz, Heidelberg, Germany), goat anti-rabbit-HRPO, goat anti-rat-HRPO, donkey anti-goat-HRPO, and rabbit anti-mouse-HRPO were acquired as secondary and/or tertiary antibodies (Dianova, Hamburg, Germany).

For pRb and p16^INK4a^ the following score was used: Staining intensity was scored by using a 1 to 4 scale: 1 = none, 2 = low, 3 = medium, and 4 = high staining intensity. A sample was considered HPV16 RNA^+^ when p16 ^INK4a^ was “high” (scores 3 or 4) and pRB was “low” (scores 1 or 2). Any other combination was considered HPV16 RNA^−^ [19]. For CD8*β* and CD45RO the following score was used: The amount of stained tissue was scored on a scale from 1 to 4: 1 ≤ 10% stained, 2 = 10–50%, 3 = 50–80%, and 4 ≥ 80% stained. The score was applied to the epithelial tumor cell nests (tumor) and to the intratumoral stroma regions adjacent to the tumor cell areas. For Fig. 7E, we combined CD8*β* and CD45RO in tumor and stroma into an “immunoscore-like grading” analogous to a recently proposed method [22]. A score of 2 or more (10% or more of tissue stained) was considered “high” for the epithelial tumor region. A score of 3 or more (50% or more of tissue stained) was considered “high” for the stromal regions. An IG0 (Immunograde 0) means that no “high” scores were assigned to the tissue; an IG4 means that four “high” scores were assigned. Immunogrades 0 and 1 were defined as “low infiltrate,” and the immunogrades 2–4 were defined as “high infiltrate” group.

IHC analysis was performed with a Zeiss Axioscope 2 microscope at 100 × and 200 × magnification (Zeiss, Jena, Germany).

#### Statistical analysis

Results are depicted as box plots with medians (and 5th, 25th, 75th and 95th percentiles shown) or scatter plots (with lines connecting samples from the same patient). Statistical analysis was performed by Mann−Whitney rank sum test, Kruskal–Wallis one-way analysis of variance followed by Dunn’s method post hoc, calculation of linear regression or Chi-squared test. A *p* value ≤ 0.05 was taken as the level of significance. The statistical analysis was performed with Systat Sigmaplot 11.0 software (Systat Software, Erkrath, Germany).

## Results

**Fig. 1:**
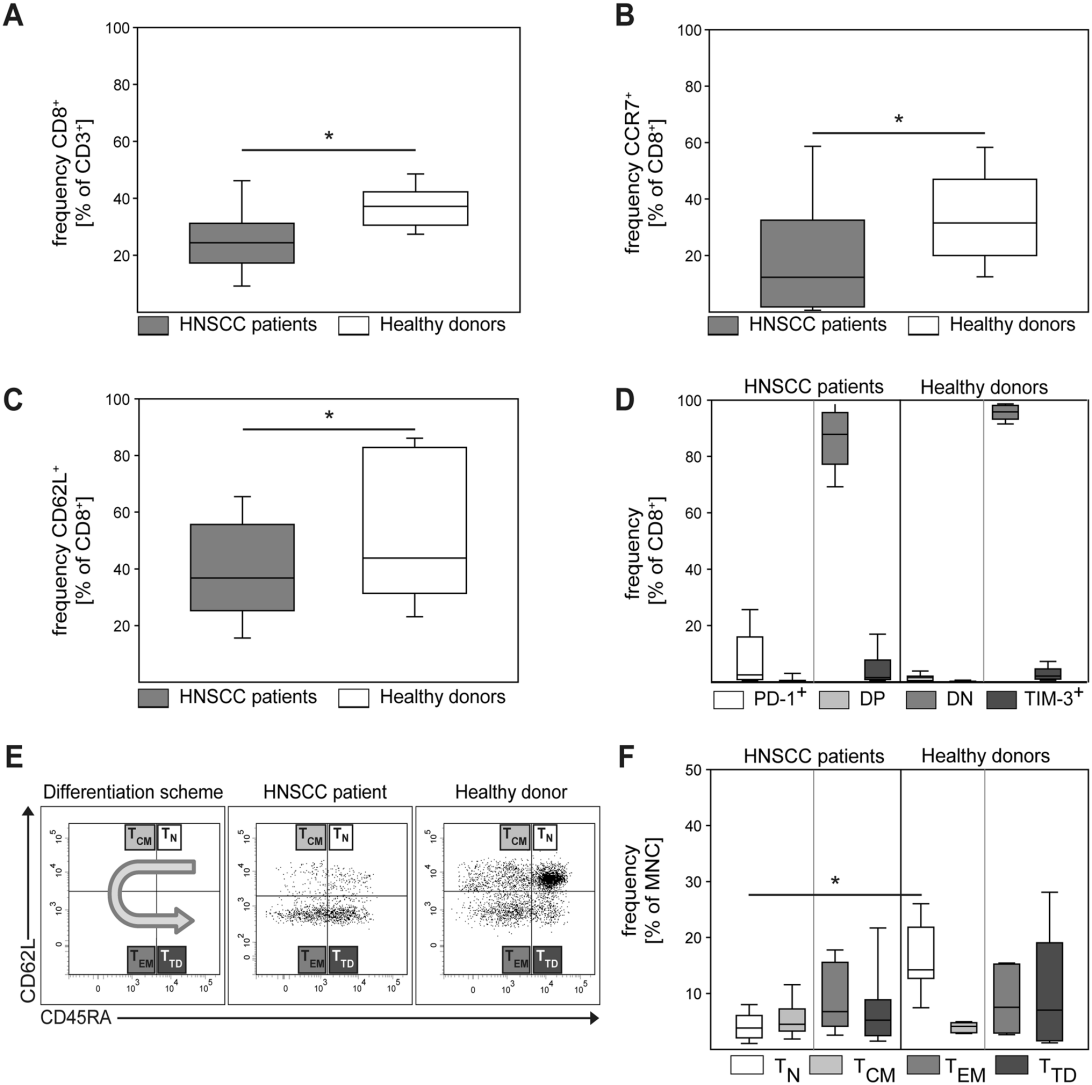
Differences in the CD8^+^ T cell compartment between HNC patients and healthy donors. PBMC from HNC patients and healthy donors were isolated by density gradient centrifugation, stored frozen until use, and analyzed by flow cytometry. Data are depicted as the relative frequency of CD8^+^ in CD3^+^ (**A**), CCR7^+^ in CD8^+^ (**B**), and CD62L^+^ in CD8 (**C**). In (**D**) expression of activation and exhaustion markers PD-1 and TIM-3 on CD8 cells was quantified by flow cytometry (DP = double positive, DN = double negative). (**E**) Depicts the gating strategy for CD8^+^ T cell phenotype subpopulations and representative examples of a HNC patient and a healthy donor. Gating was done as follows: naïve T cells (T_N_, CD45RA^+^ CD45RO^−^ CD62L^+^), central memory T cells (T_CM_, CD45RA^−^ CD45RO^+^ CD62L^+^), effector memory T cells (T_EM_, CD45RA^−^ CD45RO^+^ CD62L^−^), and terminally differentiated effector memory RA T cells (T_TD_, CD45RA^+^ CD45RO^−^ CD62L^−^). (**F**) Frequency of CD8^+^ T cell subsets in HNC patients and healthy donors. Data are presented as box plots showing median with percentiles (25th, 75th) and 5th/95th whiskers. The Mann–Whitney rank sum test (**A**–**C**) and the Kruskal–Wallis ANOVA on ranks [post hoc: Dunn ‘s method] (**D**, **F**) were used for statistical analysis. * indicates a *p* value < 0.05

Various types of squamous cell carcinoma (SCC) induce systemic immune suppression, and alterations in the T cell compartment have been reported in patients with HNC [23, 24]. Therefore, we studied a series of 89 patients with localized or locally advanced HNC treated at the West German Cancer Center. Treatment strategies (surgery alone, surgery followed by adjuvant therapy and primary radiochemotherapy) were proposed by the multidisciplinary tumor board for head and neck cancers, and treatments were conducted using state-of-the art technique and facilities (Table 1). First, we analyzed the frequency, phenotype, and subset distribution of peripheral blood CD8^+^ T cells in our cohort of HNC patients compared to healthy donors (HD). We found that the relative frequency of CD8^+^ T cells within the T cell compartment was reduced in cancer patients (Fig. 1A). CCR7, CD62L, PD-1, and TIM-3 are important markers of T cell functionality. Analyzing expression of these markers by CD8^+^ T cells, we observed a significant relative reduction of CCR7^+^ and CD62L^+^ CD8 cells in cancer patients (Fig. 1B, C). Considering the expression of activation and exhaustion markers PD-1 and TIM-3, our analysis revealed no significant differences in CD8^+^ T cells between HNSCC patients and HD (Fig. 1D), with double negative cells representing the highest fractions in both groups. We next analyzed the subset composition of CD8 cells (naïve, central memory, effector memory, terminally differentiated effector cells) in HNC patients versus HD. Following the protocol delineated in Fig. 1E we applied CD62L and CD45RA as subset markers. While the frequency of T_CM_, T_EM_, and T_TD_ was similar in HNC patients and HD, there was a striking and statistically significant reduction of naïve CD8^+^ T cells in HNC patients (Fig. 1E–F). These data support previous reports on low frequencies of CD8^+^ T cell populations in HNC patients and reveal further differences between the phenotypic T cell properties of HNC patients and HD.

**Table 1:** Clinical information for patients in cohort 1

	All patients		HPV16 DNA+ HNSCC		HPV16 DNA− HNSCC	
	Patients per group		Patients per group		Patients per group	
	*n*	%	*n*	%	*n*	%
All samples	89	100	30	100	59	100
Sex						
Male	75	84	25	83	50	85
Female	14	16	5	17	9	15
*Tumor localization*						
Oropharynx	41	46	27	90	14	24
Larynx	33	37	3	10	30	50.8
Oral cavity	6	7	0	0	6	10
Hypopharynx	3	3	0	0	3	5
Other	6	7	0	0	6	10
*T category*						
T1	18	20	4	13	14	24
T2	37	42	17	57	20	34
T3	22	25	5	17	17	29
T4	12	13	4	13	8	14
*Lymph node involvement*						
N0	40	45	6	20	34	58
N1	10	11	2	7	8	14
N2a,b	22	25	13	43	9	15
N2c, N3	17	19	9	30	8	14
*Therapy regimen*						
Surgery only	28	32	4	13	24	41
R(C)Tx	12	14	4	13	8	14
Surgery + RTx	8	9	1	3	7	12
Surgery + R(C)TX	41	46	21	70	20	34
*Human papillomavirus status*						
HPV16 DNA+	30	34				
HPV16 DNA−	59	66				
HPV16 RNA+	23	26				
HPV16 RNA−	66	74.2

**Fig. 2:**
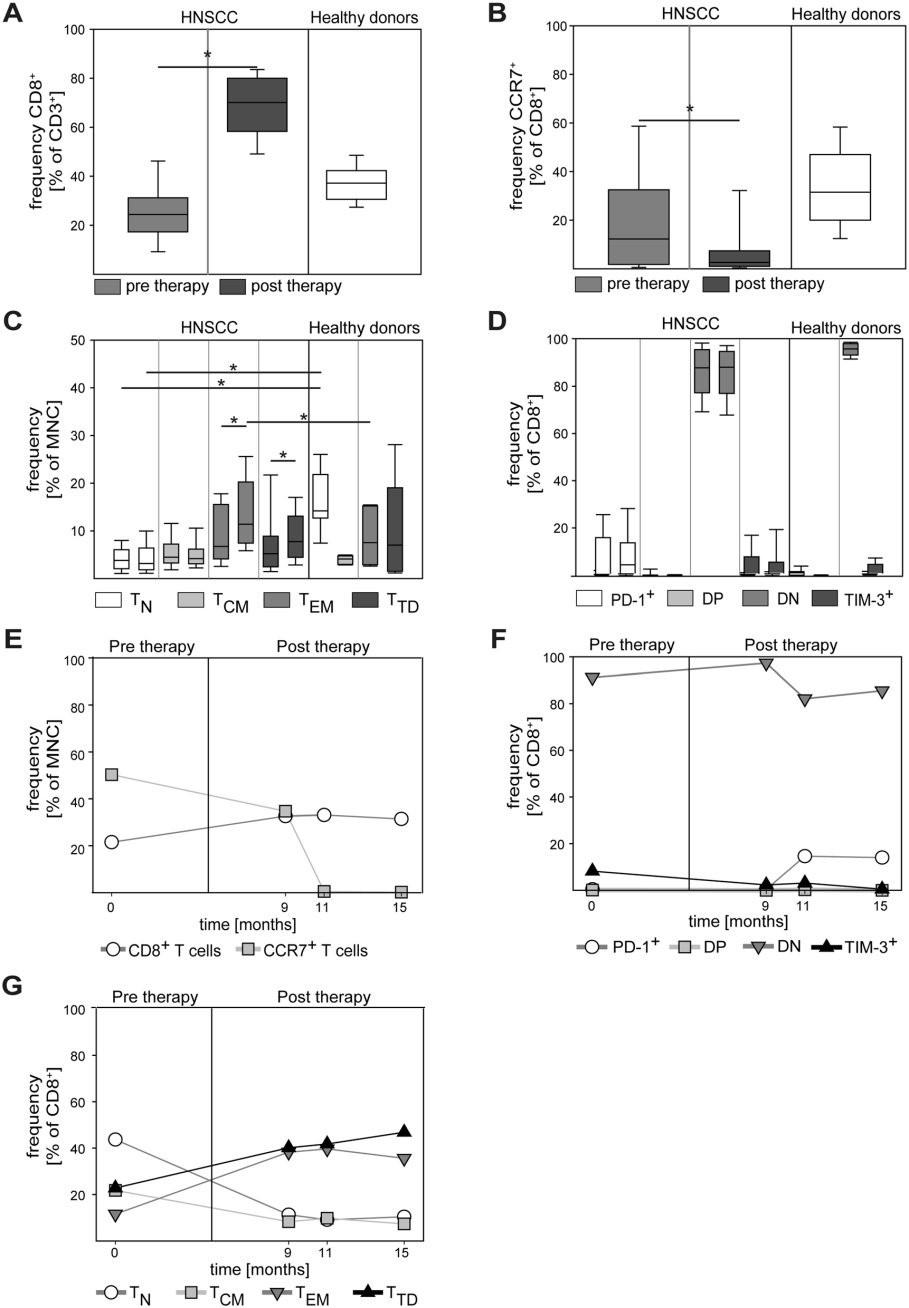
Pre-therapy to post-therapy changes in CD8^+^ T cells of HNC patients. Pre-therapy samples were obtained from tumor-bearing patients before the onset of therapy. Post-therapy samples were obtained at the first monitoring visit during routine tumor aftercare. Relative frequency of pre-therapy and post-therapy samples of CD8^+^ cells (**A**) and CCR7^+^/CD8^+^ cells (**B**) was determined in HNC patients and healthy donors. CD8^+^ T cell subsets (**C**) and expression of PD-1 and TIM-3 (**D**) were quantified by flow cytometry (DN = double negative, DP = double positive). In paired columns of the same color the left column represents the pre-therapy sample and the right column represents the post-therapy sample. Note the post-therapy increase in *T*_EM_ and *T*_TD_ in (**C**). (**E**–**G**) shows a representative example of an extended longitudinal monitoring in a HNC patient. Blood was taken over a period of 15 month. Data are presented as box plots showing median with percentiles (25th, 75th) and 5th/95th whiskers. The Mann–Whitney Rank Sum test (**A**–**B**) and the Kruskal–Wallis ANOVA on ranks [post hoc: Dunn‘s method] (**C**–**D**) were used for statistical analysis. * indicates a *p* value < 0.05

Next, we conducted a longitudinal follow-up analysis of patients and explored therapy effects on the peripheral CD8^+^ T cell compartment. As illustrated in Fig. 2A, the relative frequency of CD8^+^ T cells strongly increased in patients after therapy. Interestingly, the percentage of CD8^+^ T cells with CCR7 expression further decreased after therapy (Fig. 2B). The latter finding prompted us to investigate treatment effects on CD8^+^ T cell subsets. In agreement with the reduced expression of CCR7, we observed a significant treatment-associated relative increase in CD8^+^ T_EM_ and T_TD_ cells (left versus right columns in Fig. 2C). Surprisingly, no change in immune checkpoint expression (PD-1, TIM-3) was observed in pre- versus post-therapeutic samples (Fig. 2D). For the majority of patients, multiple post-therapeutic time points were available for analysis and representative patient trajectories suggesting durable subset shifts are shown in Fig. 2E–G.

**Fig. 3:**
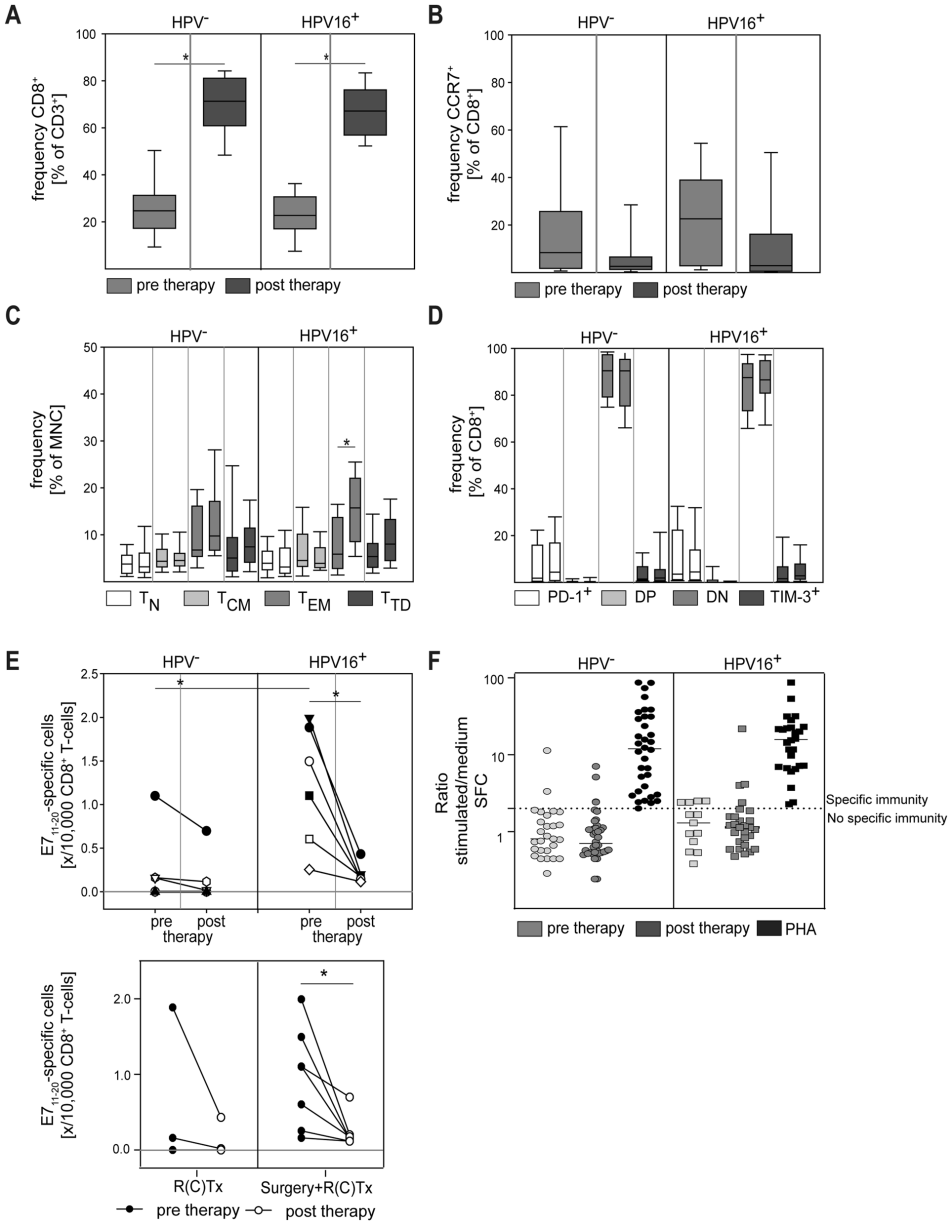
Analysis of CD8^+^ T cells in HPV^−^ versus HPV^+^ patients. HPV16 status was determined by a HPV family gene probe followed by a nested PCR to determine viral subtype if HPV^+^. The relative frequency of CD8^+^ cells (**A**) and CCR7^+^/CD8^+^ cells (**B**) was determined in pre-therapy and post-therapy samples by flow cytometry. CD8^+^ T cell subsets (**C**) and expression of PD-1 and TIM-3 (**D**) were quantified by flow cytometry (DN = double negative, DP = double positive). In paired columns of the same color, the left column represents the pre-therapy sample and the right column represents the post-therapy sample. Note the post-therapy increase of T_EM_ in HPV^+^ patients (**C**). (**E**) The frequency of HPV16 E7-specific CD8^+^ T cells was determined by flow cytometric dextramer analysis. Note the decrease of HPV-specific T cells in post-therapy samples of HPV^+^ patients. (**F**) IFN-γ ELISpot was used to determine the HPV16-specific cytokine response of CD8^+^ T cells, positive control: Phytohemagglutinin-L (PHA). Data are presented as box plots showing median with percentiles (25th, 75th) and 5th/95th whiskers. The Mann–Whitney rank sum test (**A**–**B**, **E**) and the Kruskal–Wallis ANOVA on ranks [post hoc: Dunn‘s method] (**C**–**D**) were used for statistical analysis. * indicates a *p* value < 0.05

HPV-positivity in HNC is associated with improved treatment response and better outcomes [25]. It has been suggested that improved anti-tumor immunity may contribute to this effect [26]. We, therefore, compared the treatment effect on CD8^+^ T cells and HPV-specific immune responses in HPV^+^ versus HPV^−^ patients. Therapy-associated relative increases in CD8^+^ T cells and reduction of CCR7^+^/CD8^+^ T cells could be confirmed for both HPV^+^ and HPV^−^ disease (Fig. 3A, B). However, a detailed analysis of the CD8^+^ subset composition revealed HPV-associated differences. A clear and statistically significant increase in peripheral blood CD8^+^ T_EM_ post-therapy could only be observed in patients with HPV^+^ tumors, whereas the frequency of CD8^+^ T_EM_ remained largely unchanged in patients with HPV^−^ disease (Fig. 3C). Interestingly, the frequency of PD-1^+^ and TIM3^+^ CD8^+^ T cells remained low after therapy for both patient groups. In sum, these data suggest a more pronounced treatment effect on CD8^+^ T cell phenotypes in HPV^+^ HNC.

In previous studies, T cells with specificity for HPV antigens have been reported in HPV-associated HNC. These HPV-specific T cells were mainly isolated from tissue samples [27, 28]. However, some studies reported the existence of HPV-specific T cells also in the peripheral blood, albeit at very low frequencies [29, 30]. We devised the highly sensitive dextramer technology as well as a multi-peptide ELISPOT to enumerate HPV-specific T cells pre- and post-therapy [31]. While CD8^+^ T cells with HPV16 E7-specificity could hardly be detected in HPV^−^ patients, the majority of patients with HPV16^+^ tumors displayed HPV-reactive cells at frequencies of more than 1 in 10,000 peripheral blood CD8^+^ T cells (Fig. 3E). Interestingly, associated with the removal of the primary tumor by curative therapy (primary or adjuvant radiotherapy), the frequency of these HPV16-specific CD8^+^ T cells decreased in all patients tested in our study (Fig. 3E, lower panel). Functional reactivity to HPV16 E6/E7 multi-peptides was tested by ELISPOT technology. The frequency of HPV-responsive IFN-γ producing cells was very low in pretherapeutic samples. Low-level specific responses could be detected in some patients after therapy, but no clear correlation with the HPV status was evident (Fig. 3F).

**Fig. 4:**
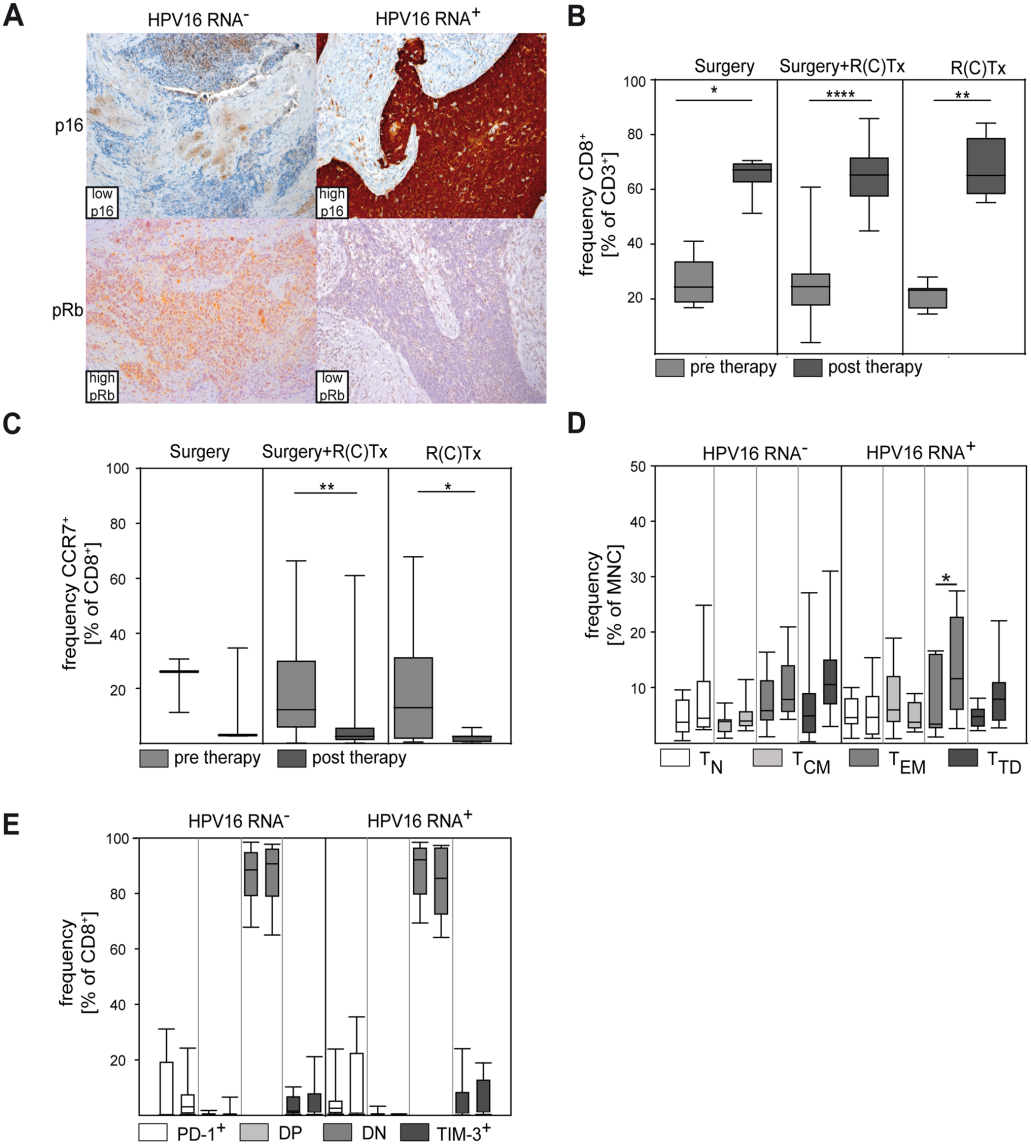
Analysis of CD8^+^ T cells in HPV16-driven versus non-HPV-driven HNC. HPV16 RNA positivity was determined by immunohistochemistry for the surrogate markers pRb and p16. Whole tissue slides (FFPE) from each patient were stained for pRb and p16 with representative examples shown in (**A**). High p16 and low pRb indicate RNA positivity and HPV16-driven HNC. Low p16 and high pRb indicate RNA negativity. The relative frequency of CD8^+^ cells (**B**) and CCR7^+^/CD8^+^ cells (**C**) was determined in pre-therapy and post-therapy (surgery, surgery + R(C)Tx, primary R(C)Tx) samples by flow cytometry. CD8^+^ T cell subsets (**D**) and expression of PD-1 and TIM-3 (**E**) were quantified by flow cytometry (DN = double negative, DP = double positive). In paired columns of the same color the left column represents the pre-therapy sample and the right column represents the post-therapy sample. Note the post-therapy increase of T_EM_ in HPV16RNA^+^ patients (**D**). Data are presented as box plots showing median with percentiles (25th, 75th) and 5th/95th whiskers. The Mann–Whitney rank sum test (**B**–**C**) and the Kruskal–Wallis ANOVA on ranks [post hoc: Dunn‘s method] (**D**–**E**) were used for statistical analysis. * indicates a *p* value < 0.05

Recent molecular analyses suggested the importance of genetically HPV-driven oncogenesis (HPV RNA positivity) compared to the presence of HPV DNA only [32]. We used a method developed by Holzinger et al. [19] as a surrogate for RNA positivity. To this end, HPV DNA^+^ samples from patients with oropharyngeal SCC were stained for p16 and pRb, and tissues with high expression of p16 and simultaneous low expression of pRb were classified as HPV16 RNA^+^ (Fig. 4A). Using this classification, no significant differences between patient stratification according to DNA positivity (Fig. 3) and HPV RNA positivity (Fig. 4) were found. Thus, our data suggest that therapy-associated changes in CD8^+^ T cells occur in both HPV DNA^+^ and RNA^+^ patients and are independent of the mode of therapy (Fig. 4B, C).

**Fig. 5:**
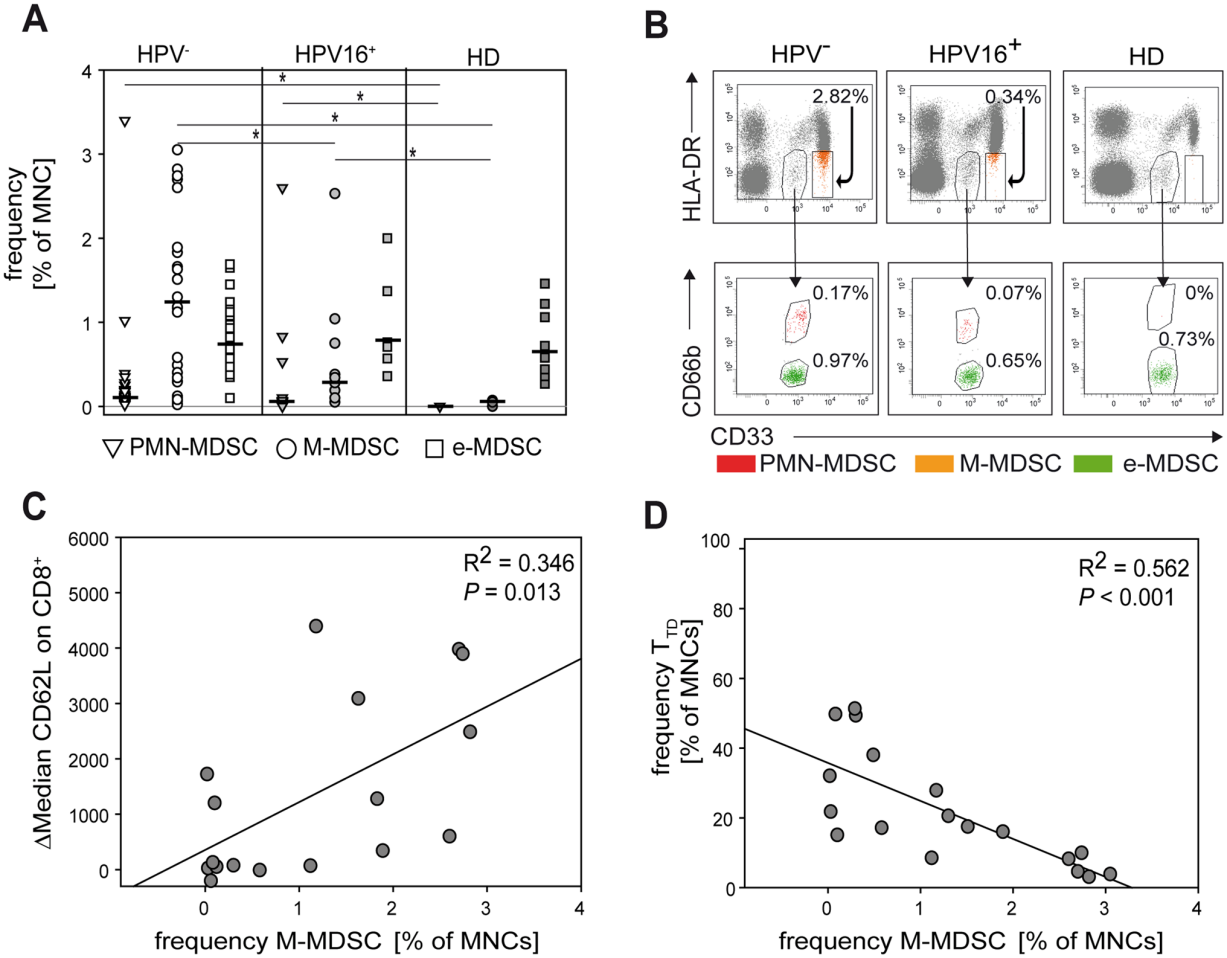
Peripheral blood MDSC frequency is linked to HPV status and correlates with T cell phenotype. MDSC analysis was performed in pre-therapy patient and healthy donor samples by flow cytometry. Polymorphonuclear MDSC (PMN-MDSC), monocytic MDSC (M-MDSC), and immature or early-stage MDSC (e-MDSC) were determined in HPV^−^, HPV^+^ HNC patients, and healthy donors. Note the increased M-MDSC frequency in HPV^−^ patients (**A**). The gating strategy is shown by representative dot plots in (**B**). Frequency of M-MDSC in HPV^−^ patients correlates with expression of CD62L (**C**, weak, yet significant) and amounts of terminally differentiated T cells (T_TD_) (moderate, **D**). Data are presented as vertical point plot or scatter dot plot with regression line. The Mann–Whitney rank sum test (**A**) and linear regression (**C**–**D**) were used for statistical analysis. * indicates a *p* value < 0.05

MDSCs regulate the activation and function of T cells in multiple murine cancer models. While the identity and function of MDSC are less clear in cancer patients, a similar function for MDSC has also been proposed in humans. Employing a previously established protocol [16, 20], we determined the frequency of the three major human MDSC subsets in the peripheral blood of our patient cohort (Fig. 5A, B). In agreement with our previous studies [8], we found an increase in both monocytic MDSC (M-MDSC) and polymorphonuclear MDSC (PMN-MDSC) in HNC patients as compared to HD. While the frequency of PMN-MDSC was similar between patients with HPV^+^ and HPV^−^ tumors, high levels of M-MDSC (above 1%) were almost exclusively detected in patients with HPV^−^ tumors. This finding was further supported by a correlation of high M-MDSC frequencies and higher expression of CD62L on CD8^+^ T cells (Fig. 5C). Also, a high number of M-MDSC moderately correlated with reduced fractions of T_TD_ effector cells (Fig. 5D). These findings establish a correlation between MDSC frequencies and CD8^+^ T cells phenotypes in HNC and suggest a link between HPV status and M-MDSC expansion. Interestingly, frequencies of all circulating MDSC subsets decreased after therapy (Fig S2) and this decrease was observed independent of anatomical tumor localization (larynx or oropharynx).

**Fig. 6:**
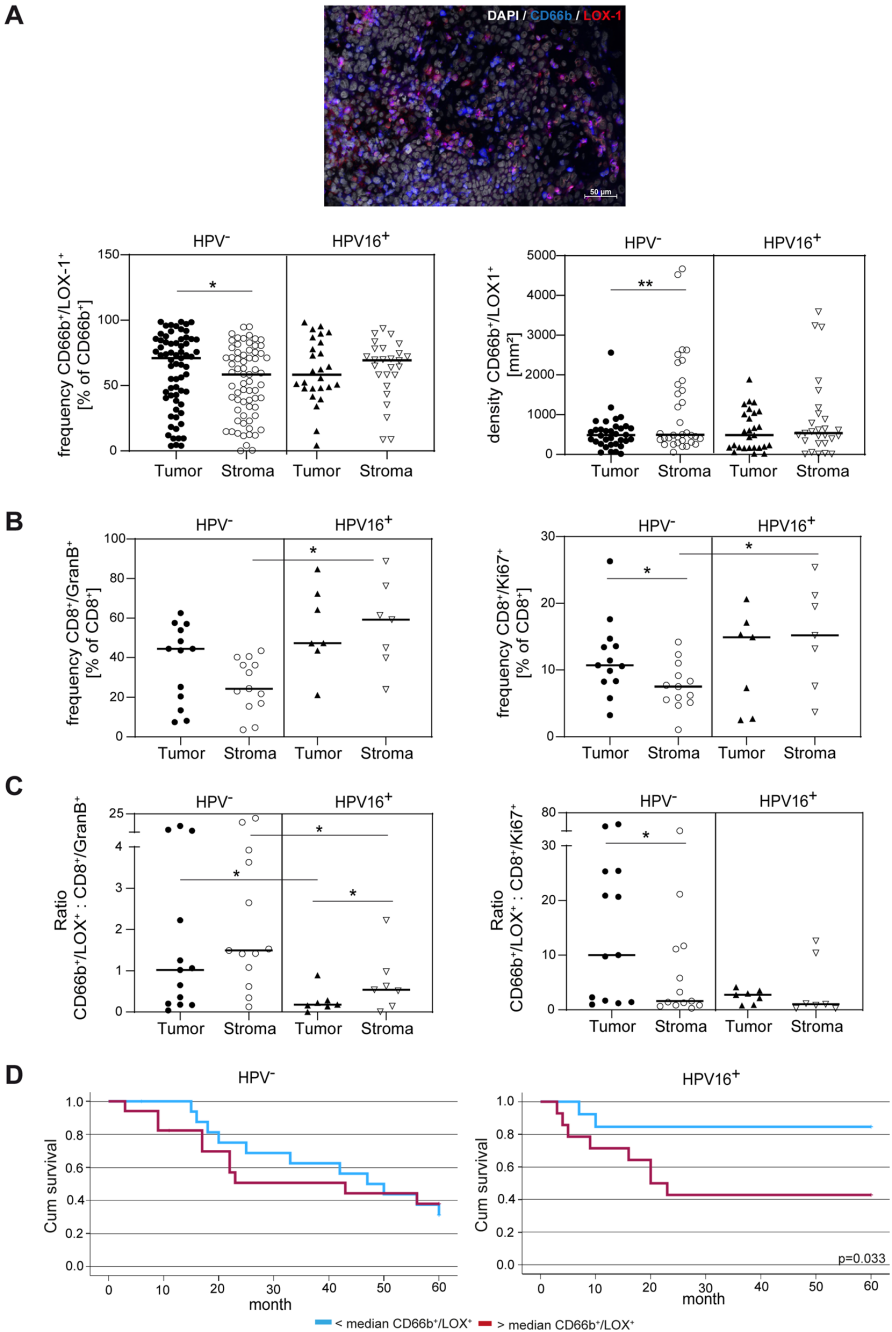
PMN MDSC fractions and CD8^+^ T cell ratios differ in HPV^+^ and HPV^−^ patients and are associated with survival in the HPV^+^ cohort. Analysis of 61 tumor tissue samples from patients with p16^+^ or p16^−^ oropharyngeal cancer (second cohort, see Table 2) by multi-color immunofluorescence. The frequency and density of CD66b^+/^LOX1^+^ PMN-MDSC (**A**) and frequency of Granzyme B^+^ cytotoxic or Ki67^+^ proliferating (**B**) CD8^+^ T cells was determined by digital pathology using tissue studio software modules. (**C**) shows PMN-MDSC to effector T cell ratios. The prognostic value of the frequency of PMN-MDSC was analyzed in HPV^+^ and HPV^−^ patients using Kaplan–Meier curves and overall survival (**D**). Data are presented as median dot plots. The Mann–Whitney rank sum test and the Kruskal–Wallis ANOVA on ranks [post hoc: Dunn‘s method] were used for statistical analysis. Survival analysis is depicted in Kaplan–Meier curves. * indicates a *p* value < 0.05

In order to support and enhance our findings from the peripheral blood, we performed an analysis of the tumor microenvironment in a second cohort (Table 2) and investigated MDSC and T cells in tumor tissue islands as well as surrounding stroma. Because no robust consensus markers for tissue M-MDSC exist, only PMN-MDSC were investigated by co-staining of CD66b and LOX1 (lectin-type oxidized LDL receptor 1) expression [14]. To further investigate CD8^+^ T cell phenotypes, Granzyme B as major cytotoxic CD8^+^ T cell molecule was chosen for analysis of T cell functionality and Ki67 as surrogate for T cell proliferation activity. Of note, the frequency and density of LOX1^+^ PMN-MDSC was increased in tumor islands of HPV^−^ patients in comparison with the surrounding stroma, a phenomenon that was not observed in HPV^+^ patients (Fig. 6A). Conversely, the prevalence of CD8^+^ T cells that express Granzyme B^+^ and Ki67^+^ was notably greater within the tumor stroma of HPV^+^ patients compared to HPV^−^ patients (Fig. 6B). Consequently, the ratio of PMN-MDSC (CD66b^+^/LOX1^+^) and proliferating (Ki67^+^) CD8^+^ T cells as well as cytotoxic Granzyme B^+^ /CD8^+^ T cells, was higher in HPV^−^ patients (Fig. 6C) suggesting a higher level of intratumoral immunosuppression in those patients. Within the HPV^+^ group, patients exhibiting low levels of intratumoral CD66b^+^/LOX1^+^ PMN-MDSC (falling below the median) demonstrated significantly improved survival rates (Fig. 6D).

**Table 2:** Clinical information for patients in cohort 2

	All patients	
Oropharynx	Patients per group	
	*n*	%
All Samples	61	100
*Sex*		
Male	38	62
Female	23	38
*T category*		
*T*1	3	5
*T*2	26	43
*T*3	20	33
*T*4	12	20
*Lymph node involvement*		
*N*0	11	18
*N*1	16	26
*N*2a,b,c	32	52
*N*3	2	3
*Therapy regimen*		
Surgery only	6	10
R(C)Tx	4	7
Surgery + R(C)TX	51	84
*Human papillomavirus status*		
HPV16 p16^+^	27	44
HPV16 p16^−^	34	56

**Fig. 7:**
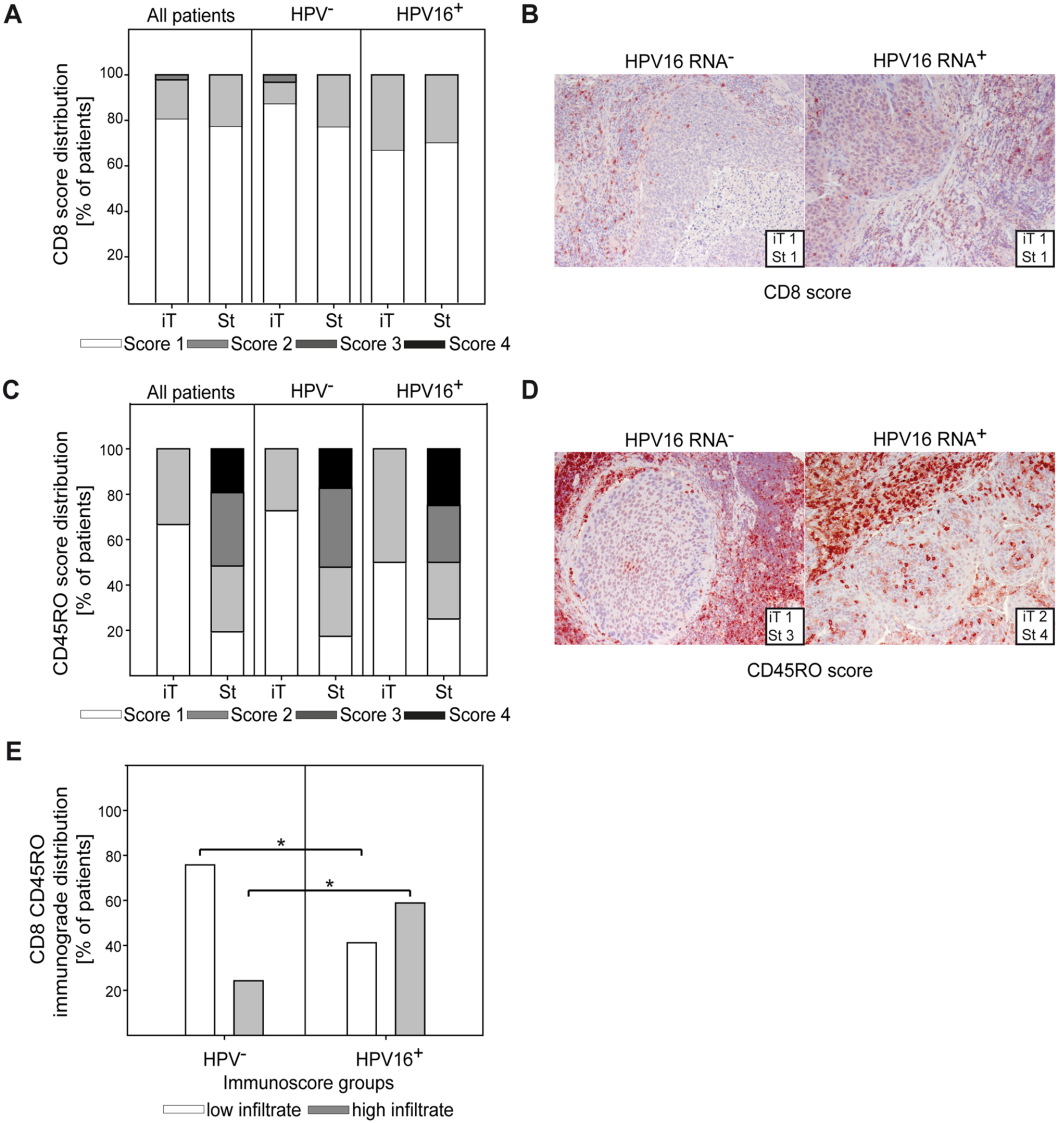
T cell infiltration in the tumor microenvironment differs between HPV^−^ and HPV^+^ patients. Tumor microarrays (including several samples for each patient) were immunohistochemically stained for CD8 and CD45RO, respectively. Epithelial tumor cell nests (iT) and intratumoral stromal (St) regions were scored independently. A score based on the approximate percentage of stained cells (1: 0–10%, 2:10–50%, 3:50–80%, and 4:80–100%) was assigned to each region by three blinded, independent, and trained investigators. Score distribution was compared between all patients, HPV^−^ and HPV^+^ HNC patients (**A**, **C**). Representative tissue slides with scores for CD8 (**B**) and CD45RO (**D**) are shown. (**E**) A combined immunograde (IG) for CD8 and CD45RO was calculated by adding the number of high infiltrate regions of CD8 and CD45RO. The score for the immunograde ranged from 0 (no high infiltrate region) to 4 (all 4 regions are highly infiltrated). IG0 and IG1 were considered the low infiltrate group, whereas the high infiltrate group consists of IG2–IG4. Data are presented as stacked box plots. The Chi-square test (**E**) was used for statistical analysis. * indicates a *p* value < 0.05

Finally, we analyzed potential differences in the CD8^+^ T cell infiltrate of HPV^+^ versus HPV^−^ tumors in patients. We focused on CD8^+^ and CD45RO^+^ cells as these markers are currently evaluated in the context of their immunoscore [33] and have been shown to be of prognostic relevance in HNC and other types of cancer [34–36]. We found that CD8^+^ T cells were evenly distributed between the epithelial tumor islands (iTu, intratumoral) and the stromal regions (St, stromal) with the majority of patients showing less than 10% CD8^+^ T cells in these regions (score 1) (Fig. 7A). CD45RO^+^ T cells were more frequent than CD8^+^ T cells and preferentially located in the stroma. Interestingly, the intratumoral region was more densely infiltrated by T cells in HPV^+^ tumors compared to HPV^−^ (Fig. 7B–D). When we combined the CD8^+^ T cell score and the CD45RO^+^ T cell score in the intratumoral and stromal region into a comprehensive “immunoscore-like grade” (see material and methods for details), an increased infiltration of HPV^+^ tumors with CD8^+^ and CD45RO^+^ cells was confirmed (Fig. 7E). Collectively, these data show a denser T cell infiltrate in HPV^+^ tumors, which is mainly caused by a stronger infiltration of the epithelial tumor islands (iT region).

## Conclusion

It has been suggested that immune-mediated mechanisms contribute to the differential prognosis of patients with HPV^+^ versus HPV^−^ HNC. In accordance with this concept, several differences between the immune status of HPV-related and non-related HNC have been described [6, 27, 37]. Herein, we carried out the first detailed longitudinal analysis of T cells and MDSC in HPV-related HNC and compared pre-and post-therapy samples from 89/61 patients from whom material was available for this type of analysis. We found that pre-therapy levels of circulating monocytic MDSC were higher in HPV^−^ disease. Importantly high numbers of MDSC correlated with low fractions of terminally differentiated CD8 effector T cells in the cohort of HPV^−^ patients. HPV^+^ tumors showed an increased infiltrate with CD8^+^/CD45RO^+^ T cells, Granzyme B^+^/CD8^+^, and Ki-67^+^/CD8^+^. This increase in CD8 effector cells was accompanied by reduced infiltration of tumor tissue by PMN-MDSC. Therapy of HPV^+^ patients was associated with an increase in CD8^+^ T_EM_ in the peripheral blood, which was not evident in patients with HPV^−^ disease.

In contrast to peripheral blood T cells, the clinical relevance of tumor-infiltrating CD8^+^ T cells is better established. A number of studies have associated high TIL counts with favorable prognosis in HNC [38–40]. Controversial data exist, however, on a potential correlation between TIL frequency and HPV status. While in some studies such a correlation was found [27], it was missing in other studies [40]. In our study we found a stronger infiltration of HPV^+^ tumors with CD8^+^ and CD45RO^+^ T cells. Considering the different biology of TIL in the tumor stroma versus tumor cell islands, we separately enumerated CD8^+^ T cells in stromal versus tumor regions and included markers originally used in the immunoscore [22]. Combining both markers and intratumoral localizations, the HPV^+^ tumors displayed a higher immune cell infiltrate. This was mainly caused by a stronger infiltration of the tumor cell islands in HPV^+^ tumors. One could speculate that this deeper infiltration into the tumor center is associated with improved anti-tumor immune activity. However, in a study by Oguejiofor only CD8^+^ stromal and not CD8^+^ T cell infiltration of the tumor area was associated with improved clinical outcome [39].

Another novel aspect of our study is the longitudinal analysis which allowed us to examine therapy-associated changes. In a previous study of 20 patients with HPV^+^ HNC, Parikh et al. found a decrease in HPV-peptide-induced IFN-*γ* in bulk cultures of PBMC when comparing pre- and post-therapy samples [41]. While the authors did not determine the frequency of HPV-specific T cells in their system, they suggested that reduced IFN-γ was caused by immune inhibitory mechanisms at post-therapy time points and induced via chemoradiotherapy. Our data show a distinct reduction of HPV-specific T cells in post-therapy samples. This reduction of HPV-specific immunity may be caused by a reduction of tumor burden and viral load. Similar observations have been made for anti-HPV serum antibodies [42]. Interestingly, in our study, this reduction in HPV-specific T cells was paralleled by an increase in the fraction of CD8^+^ T effector memory cells (T_EM_) at post-therapy time points, which is indicative of a shift in the functional, anti-tumor effective T cell compartment of these patients.

Heusinkveld et al. reported that the frequency and functionality of HPV-specific T cells are higher in the tumor as compared to the peripheral blood [28]. The exploitation of HPV-specific immunity may require therapeutic costimulation in order to achieve clinically meaningful responses [43]. Alternatively, recent advances in ex vivo expansion of HPV-specific T cells may even make adoptive T cell transfer possible [44]. We found a relatively low frequency (0.5–2 in 10.000 CD8^+^ T cells) of HPV-specific T cells in the peripheral blood of HPV^+^ cancer patients. This frequency further declined after therapy.

Interaction of PD-1 with its ligand PD-L1 has been implicated in the regulation of the immune response in HNC and other types of cancer. Our data show a low expression of PD-1 on CD8^+^ peripheral blood T cells in HNC patients. Low expression of PD-1 on peripheral blood has also been reported by Lyford-Pike et al. and by Partlova et al. [27, 45]. This is in contrast to the higher expression of PD-1 on tumor-infiltrating lymphocytes noted in those studies. Although not statistically significant in the entire cohort, we noted a small increase in the percentage of PD-1^+^ CD8^+^ T cells in the course of therapy in some patients (Fig. 2F). Originally considered an exhaustion marker, under certain conditions, PD-1 expression may also represent antigen-experienced T cells with the unique capacity to recognize tumor-specific neoantigens [46, 47]. Thus, an increase in the course of therapy, as observed in our study, could be indicative of post-therapeutic immune activation.

Recent evidence suggests that MDSC significantly contribute to the regulation of anti-tumor immunity and in particular regulation of T cell activity in murine and human malignant disease. For the first time, we comprehensively compared levels of MDSC subsets in HPV^+^ versus HPV^−^ human HNC. Interestingly the frequency of M-MDSC was significantly higher in the peripheral blood of HPV^−^ patients. In addition, in HPV^−^ disease, high levels of MDSC were associated with lower fractions of terminally differentiated peripheral blood effector T cells. In murine models of vaccination against HPV^+^ tumors, MDSC partially controlled the activity of anti-tumor CD8^+^ T cells [48]. Some earlier studies suggested that PMN-MDSC induced antigen-specific tolerance, while monocytic MDSC is involved in non-specific suppression [49]. Interestingly, in a recent multicenter analysis of circulating MDSC subsets across multiple diseases within the Mye-EUNITER (www.mye-euniter.eu) consortium, programmed death-ligand 1 (PD-L1) was found to be primarily expressed in M-MDSC [50]. As the PD-1 and PD-L1 interaction results in T cell anergy and inhibits T cell proliferation, the increased PD-L1 expressing M-MDSC fractions in HPV^−^ HNC patients potentially contribute to our observed differences in T cell phenotypes with lower T_EM_ cells and lower immunoscores for these patients. Accordingly, Spehner and colleagues identified M-MDSC levels in another HPV-dependent squamous cell carcinoma entity as the major influence on TH1-adaptive immune responses and patients' survival [51].

Additionally, our investigations show that differences between HPV^+^ and HPV^−^ tumor patients are also present in the tumor tissue. In previous work we provided evidence for in situ intratumoral activity by LOX1^+^ PMN-MDSC [14]. These findings are in line with reports by Chai and colleagues who identified ARG1/iNOS-dependent inhibition of T cell proliferation by LOX1^+^ PMN-MDSC in glioblastoma [52]. In this study we report reduced Ki67 and Granzyme B expression in CD8^+^T cells of HPV^−^ patients that also have higher PMN-MDSC / CD8^+^Granzyme B^+^ ratios. In HPV^+^ patients, we observed improved clinical outcome in patients with lower PMN-MDSC fractions. An association of MDSC and T cell frequencies was also observed by Liang and colleagues who reported—in cervical cancer patients—a negative correlation of PMN-MDSC and densities of CD8^+^ T cells, influencing clinicopathologic parameters and prognosis [53]. Although the immunoregulatory mechanisms of MDSC in cancer patients are still far from being understood, these data support a link between MDSC expansion and T cell differentiation and function, which could further explain the somewhat poor prognosis of patients with HPV^−^ disease.

In sum, our analyses revealed important differences in the MDSC and T cell compartment in HPV^+^ versus HPV^−^ disease. Patients with HPV^+^ HNC showed a post-therapeutic expansion of CD8^+^ T_EM_. These patients also demonstrated higher immune cell infiltration of their tumors as defined by the immunoscore markers CD8 and CD45RO. In contrast, patients with HPV^−^ tumors had higher pre-treatment levels of M-MDSC and this correlated with a reduced frequency of CD8^+^ T effector cells in the blood. HPV^−^ patients also showed higher frequencies of intratumoral PMN-MDSC and lower frequencies of proliferating and cytotoxic T cells. In HPV^+^ patients, lower intratumoral PMN-MDSC levels were associated with an improved survival. These differences in MDSC and T cell functionality may further contribute to and explain differences in clinical outcome of patients with HPV^+^ versus HPV^−^ HNC.

### Supplementary Information

Below is the link to the electronic supplementary material.

Supplementary file1 (PDF 449 kb)

## Data Availability

The datasets generated during and/or analyzed during the current study are available from the corresponding author on reasonable request.

## References

[1] Jung AC (2010). Biological and clinical relevance of transcriptionally active human papillomavirus (HPV) infection in oropharynx squamous cell carcinoma. Int J Cancer.

[2] Dayyani F (2010). Meta-analysis of the impact of human papillomavirus (HPV) on cancer risk and overall survival in head and neck squamous cell carcinomas (HNSCC). Head Neck Oncol.

[3] Zhang W (2016). Integrative genomics and transcriptomics analysis reveals potential mechanisms for favorable prognosis of patients with hpv-positive head and neck carcinomas. Sci Rep.

[4] Mandal R (2016). The head and neck cancer immune landscape and its immunotherapeutic implications. JCI Insight.

[5] Kimple RJ (2013). Enhanced radiation sensitivity in HPV-positive head and neck cancer. Cancer Res.

[6] Kostareli E, Holzinger D, Hess J (2012). New concepts for translational head and neck oncology: lessons from hpv-related oropharyngeal squamous cell carcinomas. Front Oncol.

[7] Lechien JR (2019). Impact of HPV infection on the immune system in oropharyngeal and non-oropharyngeal squamous cell carcinoma: a systematic review. Cells.

[8] Brandau S (2011). Myeloid-derived suppressor cells in the peripheral blood of cancer patients contain a subset of immature neutrophils with impaired migratory properties. J Leukoc Biol.

[9] Hoffmann TK (2002). Spontaneous apoptosis of circulating T lymphocytes in patients with head and neck cancer and its clinical importance. Clin Cancer Res.

[10] Strauss L (2007). The frequency and suppressor function of CD4+CD25highFoxp3+ T cells in the circulation of patients with squamous cell carcinoma of the head and neck. Clin Cancer Res.

[11] Wondergem NE (2021). At the crossroads of molecular biology and immunology: molecular pathways for immunological targeting of head and neck squamous cell carcinoma. Front Oral Health.

[12] Czystowska M (2013). The immune signature of CD8(+)CCR7(+) T cells in the peripheral circulation associates with disease recurrence in patients with HNSCC. Clin Cancer Res.

[13] Gabrilovich DI, Nagaraj S (2009). Myeloid-derived suppressor cells as regulators of the immune system. Nat Rev Immunol.

[14] Si Y (2019). Multidimensional imaging provides evidence for down-regulation of T cell effector function by MDSC in human cancer tissue. Sci Immunol.

[15] Talmadge JE, Gabrilovich DI (2013). History of myeloid-derived suppressor cells. Nat Rev Cancer.

[16] Dumitru CA (2012). Neutrophils and granulocytic myeloid-derived suppressor cells: immunophenotyping, cell biology and clinical relevance in human oncology. Cancer Immunol Immunother.

[17] Bruderek K, Schirrmann R, Brandau S (2021). Immunophenotyping of circulating myeloid-derived suppressor cells (MDSC) in the peripheral blood of cancer patients. Methods Mol Biol.

[18] Bruger AM (2020). Protocol to assess the suppression of T-cell proliferation by human MDSC. Methods Enzymol.

[19] Holzinger D (2013). Identification of oropharyngeal squamous cell carcinomas with active HPV16 involvement by immunohistochemical analysis of the retinoblastoma protein pathway. Int J Cancer.

[20] Trellakis S (2013). Granulocytic myeloid-derived suppressor cells are cryosensitive and their frequency does not correlate with serum concentrations of colony-stimulating factors in head and neck cancer. Innate Immun.

[21] Lang S (2018). Clinical relevance and suppressive capacity of human myeloid-derived suppressor cell subsets. Clin Cancer Res.

[22] Galon J (2014). Towards the introduction of the 'Immunoscore' in the classification of malignant tumours. J Pathol.

[23] Theodoraki MN (2018). Clinical significance of PD-L1(+) exosomes in plasma of head and neck cancer patients. Clin Cancer Res.

[24] Fasano M (2022). Immunotherapy for head and neck cancer: present and future. Crit Rev Oncol Hematol.

[25] Rettig EM, D'Souza G (2015). Epidemiology of head and neck cancer. Surg Oncol Clin N Am.

[26] Ferris RL, Westra W (2023). Oropharyngeal carcinoma with a special focus on HPV-related squamous cell carcinoma. Annu Rev Pathol.

[27] Partlova S (2015). Distinct patterns of intratumoral immune cell infiltrates in patients with HPV-associated compared to non-virally induced head and neck squamous cell carcinoma. Oncoimmunology.

[28] Heusinkveld M (2012). Systemic and local human papillomavirus 16-specific T-cell immunity in patients with head and neck cancer. Int J Cancer.

[29] Hoffmann TK (2006). T cells specific for HPV16 E7 epitopes in patients with squamous cell carcinoma of the oropharynx. Int J Cancer.

[30] Albers A (2005). Antitumor activity of human papillomavirus type 16 E7-specific T cells against virally infected squamous cell carcinoma of the head and neck. Cancer Res.

[31] Luckau S (2016). Vaccination against human papilloma viruses leads to a favorable cytokine profile of specific T cells. J Immunother.

[32] Mehanna H (2023). Prognostic implications of p16 and HPV discordance in oropharyngeal cancer (HNCIG-EPIC-OPC): a multicentre, multinational, individual patient data analysis. Lancet Oncol.

[33] Galon J (2014). Intratumoral immune microenvironment and survival: the immunoscore. Med Sci (Paris).

[34] Turksma AW (2013). Effector memory T-cell frequencies in relation to tumour stage, location and HPV status in HNSCC patients. Oral Dis.

[35] Wentworth L (2013). Memory T cells are uniquely resistant to melanoma-induced suppression. Cancer Immunol Immunother.

[36] Fridman WH (2011). Prognostic and predictive impact of intra- and peritumoral immune infiltrates. Cancer Res.

[37] Punt S (2016). A beneficial tumor microenvironment in oropharyngeal squamous cell carcinoma is characterized by a high T cell and low IL-17(+) cell frequency. Cancer Immunol Immunother.

[38] Jung AC (2013). CD8-alpha T-cell infiltration in human papillomavirus-related oropharyngeal carcinoma correlates with improved patient prognosis. Int J Cancer.

[39] Oguejiofor K (2015). Stromal infiltration of CD8 T cells is associated with improved clinical outcome in HPV-positive oropharyngeal squamous carcinoma. Br J Cancer.

[40] Wansom D (2012). Infiltrating lymphocytes and human papillomavirus-16–associated oropharyngeal cancer. Laryngoscope.

[41] Parikh F (2014). Chemoradiotherapy-induced upregulation of PD-1 antagonizes immunity to HPV-related oropharyngeal cancer. Cancer Res.

[42] Fakhry C (2016). Serum antibodies to HPV16 early proteins warrant investigation as potential biomarkers for risk stratification and recurrence of HPV-associated oropharyngeal cancer. Cancer Prev Res (Phila).

[43] Bartkowiak T (2015). Unique potential of 4–1BB agonist antibody to promote durable regression of HPV+ tumors when combined with an E6/E7 peptide vaccine. Proc Natl Acad Sci U S A.

[44] Ramos CA (2013). Human papillomavirus type 16 E6/E7-specific cytotoxic T lymphocytes for adoptive immunotherapy of HPV-associated malignancies. J Immunother.

[45] Lyford-Pike S (2013). Evidence for a role of the PD-1:PD-L1 pathway in immune resistance of HPV-associated head and neck squamous cell carcinoma. Cancer Res.

[46] Gros A (2016). Prospective identification of neoantigen-specific lymphocytes in the peripheral blood of melanoma patients. Nat Med.

[47] Kansy BA (2017). PD-1 status in CD8+ T cells associates with survival and anti-PD-1 therapeutic outcomes in head and neck cancer. Cancer Res.

[48] Diniz MO (2016). Protection against HPV-16-associated tumors requires the activation of CD8+ effector memory T cells and the control of myeloid-derived suppressor cells. Mol Cancer Ther.

[49] Youn JI, Gabrilovich DI (2010). The biology of myeloid-derived suppressor cells: the blessing and the curse of morphological and functional heterogeneity. Eur J Immunol.

[50] Cassetta L (2020). Differential expansion of circulating human MDSC subsets in patients with cancer, infection and inflammation. J Immunother Cancer.

[51] Spehner L (2020). Anti-telomerase CD4(+) Th1 immunity and monocytic-myeloid-derived-suppressor cells are associated with long-term efficacy achieved by docetaxel, cisplatin, and 5-fluorouracil (DCF) in advanced anal squamous cell carcinoma: translational study of epitopes-HPV01 and 02 trials. Int J Mol Sci.

[52] Chai E, Zhang L, Li C (2019). LOX-1+ PMN-MDSC enhances immune suppression which promotes glioblastoma multiforme progression. Cancer Manag Res.

[53] Liang Y (2019). Increased circulating GrMyeloid-derived suppressor cells correlated with tumor burden and survival in locally advanced cervical cancer patient. J Cancer.

