# Supplementary material for: HPV-associated head and neck cancer is characterized by distinct profiles of CD8+ T cells and myeloid-derived suppressor cells
Source: Cancer Immunol Immunother. 2023 Nov 29;72(12):4367–83. doi: 10.1007/s00262-023-03571-8 (PMC10700222; doi:10.1007/s00262-023-03571-8)
Supplement: Supplementary file 1 — Supplementary file1 (PDF 449 kb) [file 262_2023_3571_MOESM1_ESM.pdf]

Fig. S1

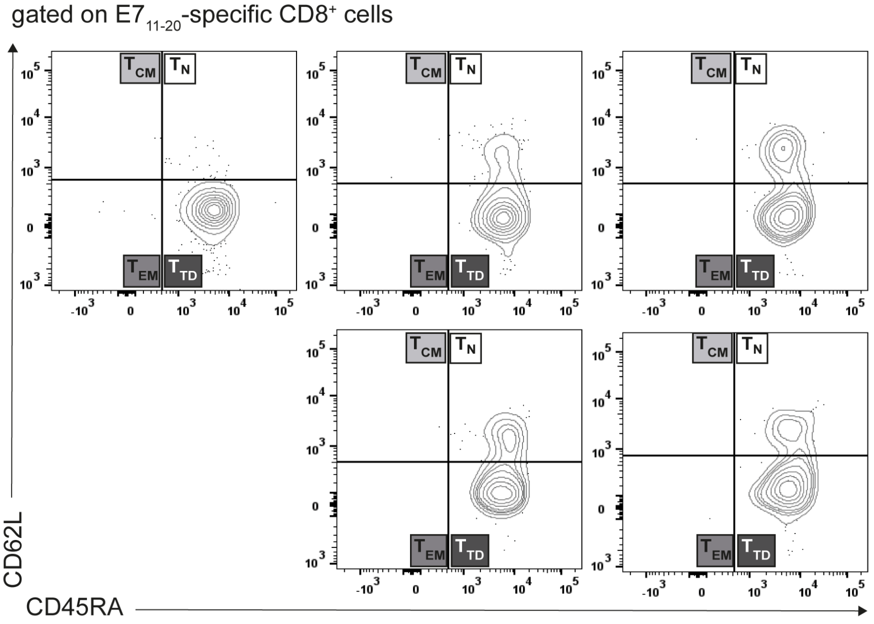

Supplementary figure S1: T cell phenotype of HPV E7$_{11\text{-}20}$ specific CD8^{+} cells

CD62L / CD45RA phenotypes of HPV E7$_{11\text{-}20}$ specific CD8^{+} cells in five different patients were stained and identified by flow cytometry

Fig. S2

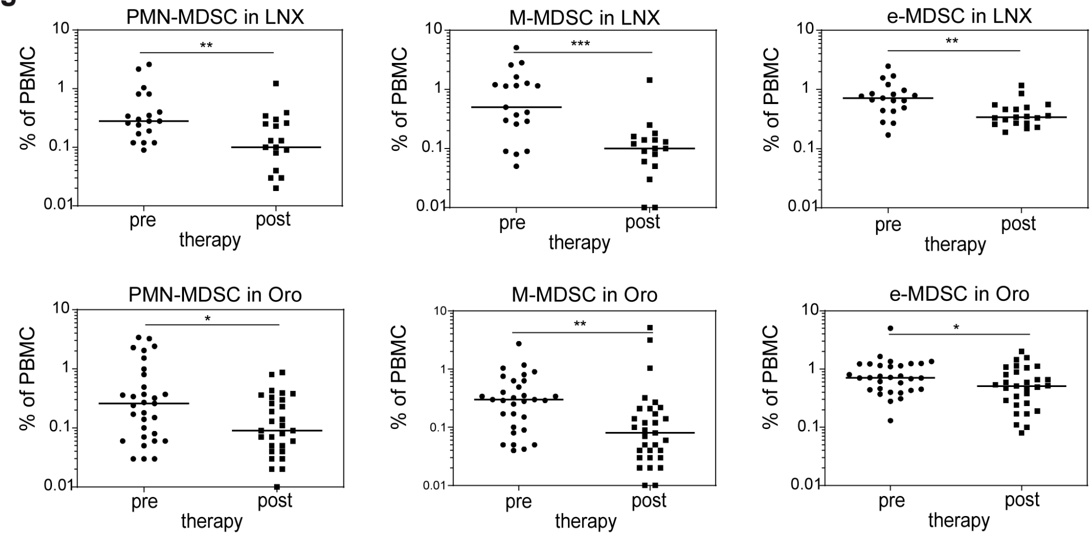

Supplementary figure S2: post-therapeutic changes in circulating MDSC frequencies according to tumor localization

The two major tumor localizations, Larynx (LNX, majority of HPV^{-} tumors) and Oropharynx (Oro, majority of HPV^{+} tumors) were investigated according to post-therapeutic changes in circulating MDSC frequencies. MDSC were identified by flow cytometry and differences between pre- and post-therapeutic frequencies are indicated.
